# Travel in the Treatment of Diseases of the Nervous System

**Published:** 1900-01-06

**Authors:** 


### TRAVEL IN THE TREATMENT OF DISEASES OF THE NERVOUS SYSTEM.

It is not improbable that one consequence of the war which has brought mourning into so many families may be a considerable diminution in the number of those who will, as a mere matter of pleasure-seeking routine, run off to warmer climates now that Christmas has passed. Many ordinary sun-seeking invalids will no doubt also be affected in the same way, and will find themselves compelled to stay at home. But on the other hand the very circumstances of the day, the anxiety, the strain, and the distress tend to the development of a large class of maladies for which the prescription of "change of scene" is commonly given, namely, functional nerve diseases. It may not, then, be out of place to consider shortly some points which arise in deciding what to do with such cases. That change of air, travel, the visiting of new places, and the arousing of new interests should have a tonic influence upon the nervous system is a fact well known to all, and is one which need not excite any wonder when one considers how intimately related are the functions of the body and the mind. Hence the frequency with which change of scene is prescribed to those whose nervous system show signs of breakdown, and the readiness with which the ordinance is obeyed. A man is said to want "shaking up," or "distracting," or what not; all these various colloquialisms merely expressing the prevailing idea that it really does not much matter where he goes, so only that he can be got "out of the rut," and one result of this common impression is that in deciding where to go the first consideration is apt to be where will he like to go? what are his tastes? and what sort of change will be most likely to perform the conjuring feat which is involved in "lifting him out of himself." It is worth bearing in mind, however, to how large an extent neuroses are but signs of bodily illness, and how frequently what appear at first as merely functional disorders of the nervous system are later seen to be the forerunners of definite organic disease, either in the nervous system or elsewhere.

It has lately been pointed out by Dr. Harry Campbell[1] that the effect of baths and climate in the treatment of nervous disorders is twofold. On the one hand, patients suffering from organic disease of the nervous system are by no means exempt from purely functional nerve disorders, and these are so much influenced for good by rest and change of scene, that although the principal malady may be thereby left untouched, the sum total of the patient's sufferings are diminished. "If, for instance, a person is hysterically inclined, the hysterical factor will tend to be brought out by such a disease as cerebral tumour or spinal meningitis, and similarly the daily variations in the symptoms of such a chronic disease as locomotor ataxy are due to variations in function which are to a large extent independent of the underlying organic disease. Now, in such cases it is no small gain to minimise the functional accompaniments, even though we are unable to touch the root disease itself, and it is here that climate and spa treatment may be of signal service to us."

On the other hand, there is the fact that nervous disorders are often secondary to disease which is not primarily nervous. In truth they are but symptoms, and the treatment required is that of the deeper lying or more general condition, and when this is relieved by climate or by the use of baths, the nervous symptoms likewise pass away.

Take, for example, so well marked a disease as chorea and its well recognised connection with rheumatism. As Sir Dyce Duckworth[2] has said, "An attack of chorea is an attack of rheumatism. . . . The clinical features of chorea indicate that the peccant matter of rheumatism has alighted in the cerebral cortex. . . . A rheumatic taint or predisposition underlies *all* cases of chorea." This being so it is not difficult to understand how essential it is in selecting a climate for such a case to bear in mind the rheumatic basis of the whole condition.

Again, a whole host of nervous disturbances are closely associated with gout in its various forms, often and perhaps more especially in cases in which its manifestations may be otherwise obscure. In such cases the gouty condition is likely to be a far more important consideration in the choice of a climate than the particular nervous disorder to which the gout has given rise. To turn such cases adrift, to be shaken up by foreign travel, is by no means a judicious procedure. Still more serious is the question of Bright's disease. The toxæmia by which this is accompanied often shows itself, as everyone knows, by its influence on the nervous system. The twitchings and nervous tricks, the headache, often taking on the form of megrim, the indigestion which may easily be mistaken for some visceral neurosis, may all seem to demand travel and change of scene for their alleviation. But if they are mere symptoms of a deep lying general disorder like Bright's disease we cannot safely give that as the prescription. For the treatment of a nervous breakdown, sight seeing in Rome or Florence may seem the very thing, but if the root malady be Bright's disease a far more protective climate may be absolutely necessary. These are but instances of a broad and general truth that nerve disease must not be considered by itself, and that in devising treatment, and especially in prescribing travel and change of air, the nature of the underlying condition is of far more importance than that of the particular nervous manifestation which may show itself.

[1] Journal of Balneology and Climatology, Oct., 1899. [2] Med. Soc. Trans., Vol. xxi.

